# Electronic monitoring of orthopedic brace compliance

**DOI:** 10.1007/s11832-015-0679-3

**Published:** 2015-08-28

**Authors:** Tariq Rahman, Whitney Sample, Petya Yorgova, Geraldine Neiss, Kenneth Rogers, Suken Shah, Peter Gabos, Dan Kritzer, J. Richard Bowen

**Affiliations:** Department of Biomedical Research, Nemours/A.I. duPont Hospital for Children, 1600 Rockland Rd, Wilmington, DE 19899 USA; Department of Orthopedics, Nemours/A.I. duPont Hospital for Children, 1600 Rockland Rd, Wilmington, DE 19899 USA; Department of Biomedical Engineering, Drexel University, 3141 Chestnut St, Philadelphia, PA 19104 USA

**Keywords:** Scoliosis, Bracing, Compliance, Electronic monitoring

## Abstract

**Purpose:**

Brace compliance measurement in adolescent idiopathic scoliosis (AIS) has been the subject of a few recent studies. Various sensors have been developed to measure compliance. We have developed a temperature-based data logger—the Cricket—specifically for scoliosis braces, with associated custom software, that is embedded directly in the brace. The purpose of this study was to analyze patterns of brace wear and patient compliance among children with AIS using the Cricket.

**Methods:**

Fifty-five AIS patients prescribed various brace-time regimens were monitored using the Cricket. All subjects were treated with the Wilmington brace. The compliance rate for each group was determined.

**Results:**

Overall compliance among subjects was 69.9 ± 31.5 %. Only 14.5 % met or exceeded prescribed brace time. This is consistent with previous compliance monitoring results.

**Conclusion:**

The results of this study objectively show the difference between prescribed and actual brace wear time and reaffirm the Cricket sensor as an accurate and comfortable brace-monitoring device.

## Introduction

Bracing is a widely used treatment for preventing progression of the curvature of the spine in adolescent idiopathic scoliosis (AIS). Bracing has been shown to be an effective treatment for AIS [1], but accurate, reliable and small sensors are needed to measure how long the brace is being worn.

Compliance has been measured using subjective means such as questionnaires [2, 3] and verbal reports [4, 5] and through objective measures like pressure monitoring [6–8] and temperature sensing [9–12]. However, these sensors have primarily been used in research studies and have not been part of the clinical service provided with the brace. The Bracing in Adolescent Idiopathic Scoliosis Trial (BRAIST), a large-scale, multi-institutional study that included a randomized and preferential treatment group, used temperature loggers embedded in the brace to monitor wear time [1]. They found a significant benefit to brace wearing compared to the observational group, as well as a correlation between brace wear time and success rate [13]. The sensor used in the BRAIST study was an off-the-shelf sensor that was post-processed for the compliance data.

In previous studies [14–16], an electronic compliance monitor (Creative Micro Designs, Newark, DE, USA) was shown to objectively measure brace wear compliance for patients with AIS while addressing some shortcomings in the old sensors, such as bulkiness, difficulty attaching to the brace, download difficulty, fragility, risk of water damage, and lack of an LCD readout for daily feedback to the patient and family. The sensor was shown to be 98 % reliable when compared to the gold standard of timed brace wear. In this study the Cricket sensor was used to look at patterns of brace wear among patients with idiopathic scoliosis during routine clinical visits.

## Materials and methods

After an Institutional Review Board approved this study, 55 patients (three boys and 52 girls) with AIS from the existing outpatient clinics at Nemours/Alfred I. duPont Hospital for Children agreed to participate. Data were collected from September 2009 to November 2011. The inclusion criteria in this study were patients with AIS treated with the Wilmington scoliosis brace. The patients were aged 10 years or older, with a Risser sign of 0–2, curves of 25°–40° and no prior treatment, consistent with the optimal inclusion criteria outlined by Richards et al. [17]. Exclusion criteria were non-idiopathic scoliosis. Prescribed regimens for brace wear time ranged from 8 to 24 h per day depending on the age and severity of the scoliosis. The number of hours prescribed (number of patients) was as follows: 8 (2), 10 (2), 12 (25), 18 (14), 20 (5), 23 (4), 24 (4). Patients participated in one to four trials each. A trial consisted of the brace-wearing period between the clinical visits. A total of 95 trials were recorded.

**Fig. 1:**
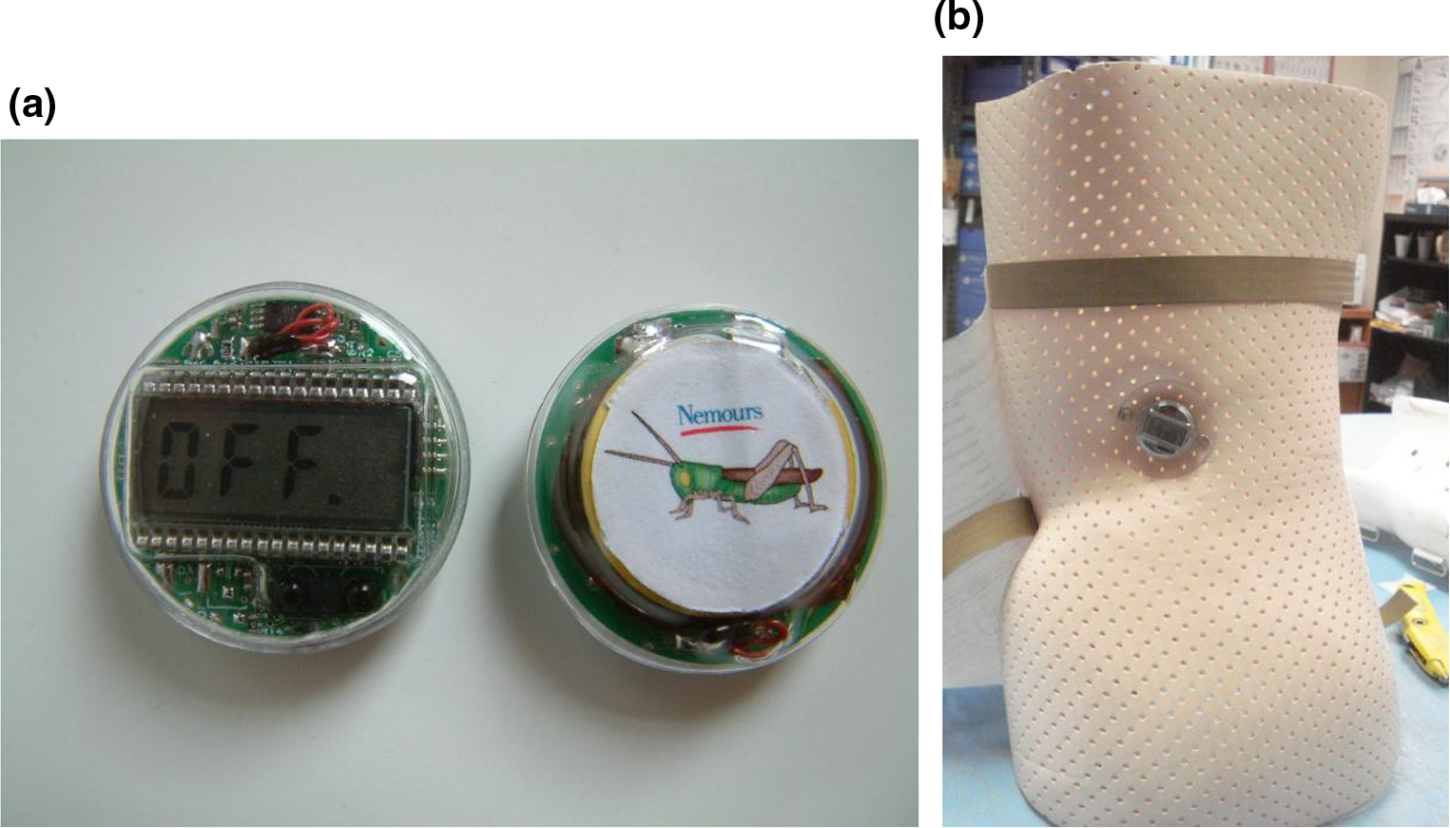
**a** The Cricket shown with the LCD display, **b** mounted in the lumbar mold of the scoliosis brace

The Cricket (Fig. 1), a small sensor designed specifically for the purpose of measuring compliance of AIS brace wearers, was embedded in the side of each patient’s custom-made Wilmington scoliosis brace. Every 10 min throughout each trial, the device took and stored a temperature reading. At each patient’s appointment, data were downloaded through an infrared reader and analyzed by custom-designed software. A threshold temperature was set in the software to indicate body temperature. All readings above this threshold corresponded to skin temperature and the brace was considered ‘worn’. Readings below the threshold corresponded to ambient temperature and were considered ‘brace not worn’. In this way, the software calculated the number of hours the brace was worn each day. Compliance—the ratio of measured brace wear time to prescribed brace wear time—was calculated from these data. The Cricket sensor has been shown to be 97 % accurate when compared to the gold standard of a diary [15]. Further details on the Cricket design can be found in Rahman et al. [16].

## Results

**Fig. 2:**
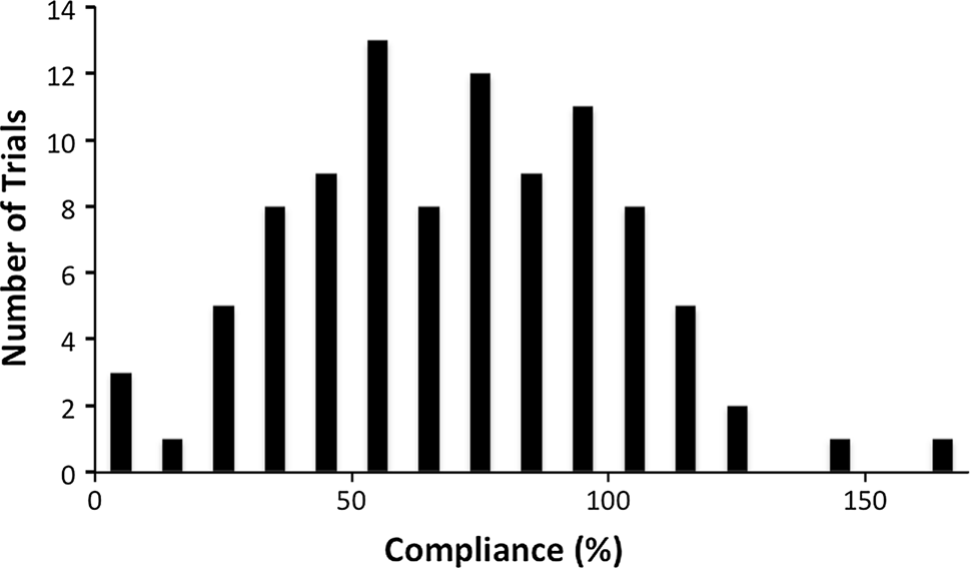
Frequency distribution of number of trials versus compliance percentage. A trial signifies one time period between clinic visits. The Gaussian characteristic is indicative of a normal population distribution

Seventy-three patients were recruited for the study. Eighteen subjects did not complete the study: six dropped out (five due to surgery, one had pain using the brace), three had improperly consented and were therefore excluded, and in nine the battery died. Fifty-five subjects completed the experiment. Overall compliance for all trials was 69.9 ± 31.5 % (mean ± SD), and the frequency distribution was similar to a Gaussian distribution (Fig. 2).

Actual hours worn per day and compliance percentage varied among the patients and trials. The compliance rate was obtained from the following formula: (sensor wear time × 100)/prescribed time.

**Fig. 3:**
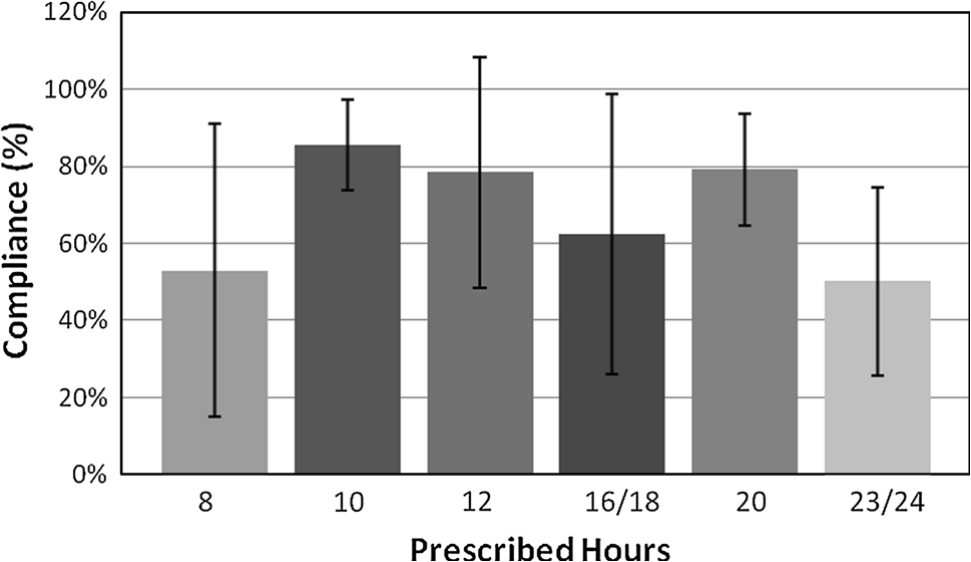
Compliance rates of patients at prescribed brace-wearing times. There is no correlation between compliance rates and prescribed wear time, and there is a large variability regardless of the prescribed wear time

The average number of days for each trial was 112.45 ± 65.35. The average daily wear time was 10.70 ± 5.22 h. There was no correlation between prescribed time and compliance (Fig. 3). Of the 55 patients, 14.5 % (eight patients) met or exceeded 100 % compliance, while 85.5 % (47 patients) did not fully comply with their prescribed time. Additionally, 14.5 % (eight patients) reached at least 90 % compliance, and 65.5 % (36 patients) reached at least 50 % compliance.

## Discussion

Bracing is a commonly used non-operative treatment for AIS, and has been shown to be effective in decreasing the progression of high-risk curves [13, 18–23]. However, it has not clearly been established how often bracing works, which curves are correctable by brace, and how long braces need to be worn to be effective? In order to study these issues, an objective method of measuring compliance to prescribed brace usage may be helpful.

The Cricket thermal sensor was developed to accurately measure the number of hours the brace is worn each day [14, 16]. The Cricket is small enough to be embedded in a standard brace so that the surface of the brace is smooth and contoured. It is both shock- and water-proof to reduce the risk of damage during everyday wear. An LCD readout faces the outside of the brace and provides information on daily wear and accumulated usage [16].

In this study, we found that brace-wearing compliance followed a normal distribution, based on a chi-squared goodness-of-fit test (Fig. 2). Overall compliance for all trials was 69.9 ± 31.5 %. These results are consistent with previous compliance measurements determined by Takemitsu et al. (75 ± 27 %; unpaired 2-tail *t* test; *p* = 0.29) [14]. Compliance data from the initiation of brace wear were included. This may result in underestimation of the compliance rate to account for the initial period of getting used to the brace when patients may wear it less. The variance is quite large, which may be due to the relatively small number of subjects and the variability of the prescribed times. A shortcoming of this study is that we did not have a control group that were blinded to the use of the Cricket. This may lead to an overestimation of the compliance rate as the blinded group may be less compliant [24].

During the course of this study, we came across several areas in which the design of the Cricket could be improved. Several subjects were unable to complete the study because of inadequate battery capacity. We recommend using a battery with more capacity. Additional memory would also be beneficial so patients could be tracked over a longer period of time; currently the memory is full at about 9 months of recording. This is adequate for most of the patients as they come back to the clinic prior to 9 months.

In addition to the benefits of obtaining accurate compliance data for research purposes, there are several possible benefits of including this device in all scoliosis braces. First, the LCD readout provides useful information about compliance to both the patient and the parent. Patients in a standard brace may forget how long they have been wearing it or intentionally mislead their parents to avoid having to wear an uncomfortable brace for the full prescribed time. With the LCD readout, patients can easily check how close they are to the prescribed time and parents can encourage brace wearing in non-compliant children. Parents in this study reported that the LCD feature was useful in this regard, though further studies are needed to confirm this.

Similarly, patients may be more likely to wear the brace for the fully prescribed time if they know they are being monitored. Because patients know that their physicians can track their wear patterns, they might be more compliant in order to avoid criticism. In future, perhaps the physician could be alerted if compliance decreased below a certain threshold, prompting them to contact a parent or guardian.

## Conclusions

The results of this study objectively confirm that wear times largely fall short of prescribed times, regardless of the prescribed wear time. We have shown the Cricket to be an effective compliance monitor for scoliosis brace wearing in clinical and research settings. It can be a valuable tool in parental monitoring and increasing brace compliance.
